# A Versatile and Modular Microfluidic System for Dynamic Cell Culture and Cellular Interactions

**DOI:** 10.3390/mi16020237

**Published:** 2025-02-19

**Authors:** Qasem Ramadan, Rana Hazaymeh, Mohammed Zourob

**Affiliations:** 1 College of Science & General Studies, Alfaisal University, Riyadh 11533, Saudi Arabia; 2 College of Pharmacy, Almaarefa University, Riyadh 13713, Saudi Arabia; rhazaimeh@um.edu.sa

**Keywords:** organ-on-a-chip, microfluidics, fabrication, integration

## Abstract

A versatile and modular microfluidic system for cell co-culture has been developed. Microfluidic chips, each featuring dual compartments separated by a porous membrane, have been fabricated and assembled within the system to facilitate fluidic interconnection and cell–cell communication through the chip assembly. A set of fluidic valves has been successfully integrated to regulate the flow through the chip assembly. The system allows for chip assembly in various arrangements, including in parallel, in series, and complex connections. Individual chips can be interconnected or disconnected within the system at any time. Moreover, the spatial order and orientation of the chips can be adjusted as needed, enabling the study of different cell–cell arrangements and the impact of the presence or absence of specific cell types. The utility of the system has been evaluated by culturing and interconnecting multi-monolayers of intestinal epithelial cells as a model of the complex cellular system. Epithelial monolayers were grown in multiple chips and interconnected in various configurations. The transepithelial electrical resistance and permeability profiles were investigated in detail for these configurations upon treatment of the cells with dextran sulfate sodium. Immune cells were stimulated through the epithelial layers and the expression of inflammatory cytokines was detected. This miniaturized platform offers controlled conditions for co-culturing key cellular components and assessing potential therapeutic agents in a physiologically relevant setting.

## 1. Introduction

Considerable research endeavors are presently focused on exploring approaches to shift the process of drug screening and toxicity testing from a reliance on animal studies to primarily utilizing human-relevant in vitro models. This shift aligns with the evolving regulations that discourage the use of animal testing and acknowledges the inherent disparities between animal and human systems [1,2]. The development of satisfactory in vitro approaches for predicting drug response in humans has yet to be achieved. The current in vitro drug screening tools fail to replicate the complexity of the human system. Additionally, they rely on studying the effects and toxicity of drugs using a single cell type, which does not accurately reflect the multi-cellular structure found in vivo. As a result, these models are unable to capture the complex interactions that occur under pathophysiological conditions over extended periods of time. Moreover, the use of these methods necessitates a large quantity of cells, reagents, and culture media, making them quite costly. Furthermore, they fail to create a dynamically controlled cell microenvironment, thereby limiting their physiological relevance. Consequently, there is a real need for developing in vitro models of human organs that closely replicate the physiological processes involved in disease development. These models should possess a desirable level of flexibility, accuracy, and reproducibility to facilitate efficient screening of potential drug candidates. Microfluidic-based organotypic cell culture models have emerged as promising tools for creating an “in vivo-like” cell microenvironment. These models offer greater feasibility for automation and integration, making them efficient tools for disease modeling and drug screening [3,4,5,6,7,8,9].

In the body, tissues and organs are engaged in communication through the secretion of a diverse array of soluble factors and extracellular vesicles. These molecules play a vital role in facilitating communication with the circulatory system and peripheral crosstalk. In an in vitro setting, the interconnection of different organ modules has a profound impact on their functionality and effectiveness [5,10,11]. To study inter-organ communication, a common medium that mimics circulating blood is commonly used to transport essential components such as nutrients, soluble factors, cell metabolites, and drugs. Development of a universal cell culture medium that effectively maintains the phenotypes and functions of all organs within an in vitro system presents a significant challenge. It is worth noting that this approach is primarily applicable to mature tissues that exhibit phenotypic stability.

To create an in vitro organotypic cellular structure that facilitates the exchange of fluids, biochemicals, and chemicals, microfluidic systems are engineered. These systems enable the precise arrangement of various cell types, mimicking the architectural organization of tissues observed in vivo [10,12]. Organ-on-a-chip (OOC) technology represents a sophisticated approach for designing cell culture systems. It offers means to achieve precise cellular positioning and in vivo-like cell polarization by providing a template where cells can reproduce a complex assembly and mimic the organization found in actual tissues [13]. Multicellular structures such as spheroids or organoids can be achieved by employing 3D cell culture techniques [14]. However, in vivo tissue architectures do not typically form naturally in spheroidal structures. Therefore, specific cell culture methods need to be used to induce the formation of cellular assemblies and their associated extracellular environment. One example of such architectural features in cellular organizations is polarity, which represents an inherent characteristic of epithelial cells [15]. The establishment of polarity is facilitated by the asymmetrical distribution of proteins across the cell membrane, a process regulated by the formation of tight junctions (TJs) between neighboring cells [16]. These TJs serve as a barrier between the basolateral and apical membranes, playing a pivotal role in the paracellular absorption process [17,18]. Microfluidic-based cell culture allows seamless integration of diverse cell types and organs within a unified fluidic circuit. This innovative approach enables the establishment of organ–organ crosstalk, while simultaneously preserving the individual functionality of each organ. Additionally, this technology could faithfully replicate the crucial role of vascular perfusion found in vivo.

The design of OOC systems is primarily guided by the physiological parameters that need to be replicated in an in vitro setting [19]. This can be accomplished by creating a minimal functional structure of an individual organ or by integrating multiple organs within the system [20,21,22]. Key cell models, selected from either cell lines or induced pluripotent stem cell (iPSC) sources, are utilized to represent the specific organ or organs of interest [23,24]. In addition, biochemical stimuli such as drugs and toxins, as well as physical stimuli like hydrodynamic, mechanical, and electrical cues, are employed to simulate the relevant physiological conditions within the OOC system. OOC systems facilitate dynamic interactions between diverse cell types, thus allowing for the replication of specific functions in an in vitro setting with the ability to conduct time-resolved measurements at various checkpoints, thereby providing insights into dynamic biological processes.

The successful implementation of OOC devices relies on the design of a compartmentalized fluidic system, which is instrumental in establishing a relevant and effective in vitro model [25]. This design framework necessitates careful consideration of various critical parameters. Among these, determining the appropriate size of individual compartments is of paramount importance, as it should accurately correspond to the size of the respective organ. Furthermore, the sequence in which the organs are interconnected, the orientation of the tissue, and the perfusion rate within each compartment which are essential to ensure an optimal design must be carefully considered.

To investigate the impact of inter-organ communication, it is essential to create multiple tissue environments. In vivo, isolating the interactions between two organs presents challenges due to their placement within the whole-body milieu. Signals released by each organ rapidly disperse into the bloodstream, reaching numerous other tissues, making it challenging to ascertain the relative contributions of each cell type to specific physiological events [26].

**Figure 1:**
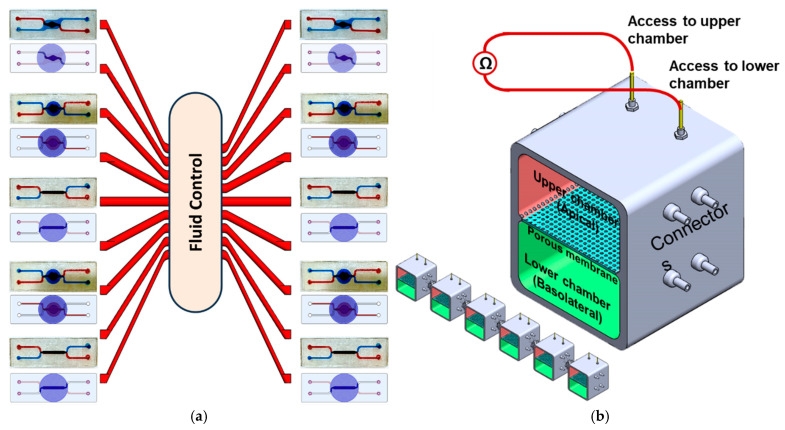

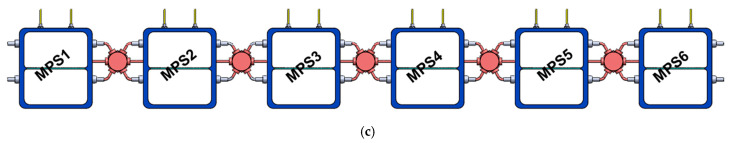
(**a**) Linking multi-organ models in one fluidic system. Each organ model can be hosted in an individual chip, and the chips can be fluidically connected to allow paracrine/endocrine signaling, mimicking the organ–organ crosstalk in vivo. (**b**) Schematic representation of the system with six chips connected through a set of valves. An enlarged view of a single chip which comprises two chambers (upper/apical and lower/basolateral) separated by a porous membrane. (**c**) 2D view of the connected chips, each hosting an in vitro model with the valving modules that enable custom linking of two or more chips in various configurations.

Here, we describe a modular microfluidic system that can be utilized to grow different types of cells in various two/three dimensional (2D/3D) architectures with improved cell–cell/tissue–tissue interaction, hence enabling the recapitulation of the structure of an individual human organ or a network of organs in vitro. The microsystem allows for real-time monitoring of biological barriers such as the intestines, which serve as crucial gateways for drug delivery. This monitoring is achieved through the utilization of a set of micro-electrodes. This platform embodies a controlled miniaturized system that allows for the simultaneous presence of key cellular components and the evaluation of potential therapeutic agents within a physiologically relevant environment. Figure 1 provides a schematic conceptual illustration of the system.

## 2. Materials and Methods

### 2.1. Materials

Poly(methyl methacrylate) PMMA sheets with thicknesses of 0.5 mm, 1 mm, and 2 mm were purchased from a local vendor. Double-sided adhesive (3M 467MP Adhesive Transfer Tape Acrylic 2.3 mil) is from 3M (Saint Paul, MN, USA). 3D printing resin (DentaGuide) is from Asiga (Alexandria, NSW, Australia). Human colorectal adenocarcinoma cells (Caco-2) are from the American Type Culture Collection (ATCC) (Manassas, VA, USA). Poly-L-lysine (PLL) solution, 0.1% (*w*/*v*), collagen (type 1, rat tail), and Trypsin/EDTA were purchased from Sigma-Aldrich (Burlington, MA, USA). Dulbecco’s Modified Eagle Medium/Ham’s F-12 (DMEM/F12) containing 10% (*v*/*v*) fetal bovine serum and 1% penicillin–streptomycin antibiotic was purchased from Gibco; TNF-α, IL-6, and IL-8 ELISA kits were purchased from Thermo Fisher Scientific (Waltham, MA, USA); Hoechst 33342 was purchased from Molecular Probe (Eugene, OR, USA); Bradford reagent and 0.4% trypan blue were purchased from Sigma-Aldrich (Burlington, MA, USA).

### 2.2. Chip Fabrication

**Figure 2:**
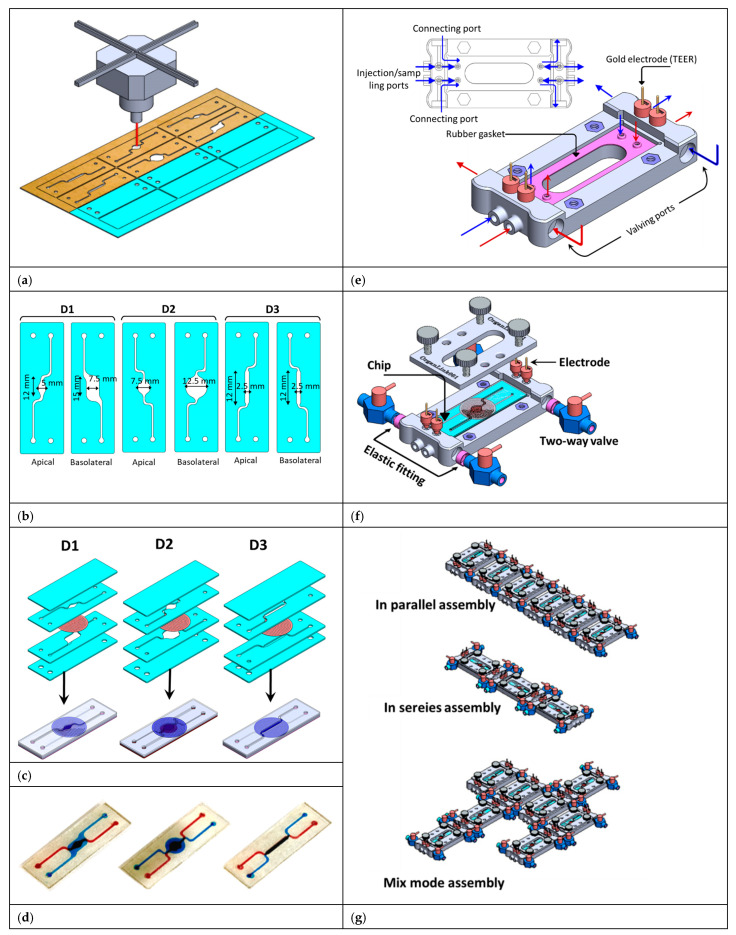
(**a**) The microfluidic chip was fabricated using PMMA using laser machining. Before cutting, one half of the PMMA sheet was laminated with a double-sided adhesive tape to facilitate the bonding of the four layers together. (**b**) Top view of the four layers with the fluidic chambers’ dimensions. (**c**) The assembly of the four layers with the porous membrane to form the chips. (**d**) Optical images of the assembled chips with colored liquid injected to enhance visualization. (**e**) The design of the fundamental unit incorporates multi-directional fluidic connection gates and integrated electrodes for measuring TEER. (**f**) A detailed view of the base unit with the chip inserted in. (**g**) Three different chip assemblies: in parallel, in series, and mix mode.

The fabrication of the system relies on multiple microfabrication processes to realize a manufacturable prototype. The 3D microfluidic chips were fabricated using the laser machining technique and adhesive-based bonding (Figure 2a). Three chips (called D1, D2, and D3) with different configurations (i.e., different chamber dimensions and shapes) were designed using AutoCad software (2022) (Autodesk, San Francisco, CA, USA) (Figure 2b) and the file was then transferred to the laser cutter (Beambox, Flux, Taipei, Taiwan) using the Beam Studio software (Version 1.9.5) (Beambox, Flux, Taipei, Taiwan). The 3D microfluidic devices were realized by bonding four layers of polymethyl methacrylate (PMMA) and a track-etched polyester (PET) membrane (iPCELLCULTURE, it4ip, Louvain-la-Neuve, Belgium) to form a fully sealed 3D microfluidic chip with vertically stacked chambers sandwiching a porous membrane with a pore size of 0.4 μm and a thickness of 10 μm (Figure 2c). For bonding and chip assembly, a PMMA sheet with a thickness of 1 mm and dimensions of 50 × 30 cm^2^ was laminated on both sides with a double-sided adhesive tape, with a thickness of ~150 µm, and cut with the laser machine to fabricate the two inner layers (Figure 2c). Another un-laminated PMMA sheet was used to cut the outer layers (Figure 2c). Then, the four layers and the porous membrane were assembled with the order shown in Figure 2c to form the chip. The resultant chip comprises two chambers (upper/apical and lower/basolateral) separated by a porous membrane. The fluidic volumes of the lower chambers are 112.5 µL, 122.6 µL, and 30 µL for D1, D2, and D3, respectively, while the volumes of each of the apical chambers are 39 µL, 44.15 µL, and 30 µL for D1, D2, and D3, respectively. Figure 2d shows the fabricated chips with colored liquid injected in the chambers to enable visualization of the fluid flow and fluid diffusion from the apical chamber to basolateral chamber through the membrane.

It is important to note that, although the chip dimensions produced in this study exceed the microscale (i.e., >100 µm), they are well suited for cell culture applications. Microfluidic devices are engineered to control and examine small volumes of fluids, typically ranging from microliters to nanoliters. The absence of a precise definition regarding the size range of microfluidic devices stems from the diverse applications and designs within this field. These devices find utility in various domains, including chemical synthesis, biological analysis, and drug delivery, with their dimensions adapting to meet the specific requirements of each application. Therefore, we have chosen to employ the term “microfluidic” instead of “millifluidic”, as the latter is not widely recognized or commonly used within the field.

### 2.3. Fabrication of the Fluid Routing Unit

The fluid routing unit (FRU) plays dual roles as a jig for facilitating cell seeding, fluidic injection, and sampling and as a platform for liquid exchange between various adjacent chips. The base unit (chip holder) of the FRU holds a single chip within a cavity with length, width, and depth of 75 mm, 25 mm, and 2 mm, respectively. The chip holder comprises 8 fluidic ports and a channel network for introducing and extracting cells and liquid into and outside the chip as well as linking the next chip (Figure 2e,f). The base unit comprises four ports which enable inserting the connecting valves (Figure 2e,f). These valves allow connecting/disconnecting the adjacent chips for inter-chip fluidic routing. Figure 2g show the modular FRU with 6 base units connected in series, in parallel, and complex connection configurations. The FRU was designed using SolidWorks software (Dassault Systèmes, Vélizy-Villacoublay, France) and fabricated using the 3D printing technique using an ASIGA MAX UV 3D printer (ASIGA, Alexandria, NSW, Australia) and employing the Asiga DentaGUIDE biocompatible resin. The base units within the FRU were connected through a set of two-way valves/sample injection/shut-off valves (Dawin Microfluidics, Paris, France) with a channel diameter of 0.5 mm. Also, each base unit was integrated with four gold wires for measuring the transepithelial electrical resistance (TEER) with two electrodes accessing the apical chamber and two electrodes accessing the basolateral chamber (Figure 2e–g).

### 2.4. Chip Assembly and Inter-Chip Fluid Routing

**Figure 3:**
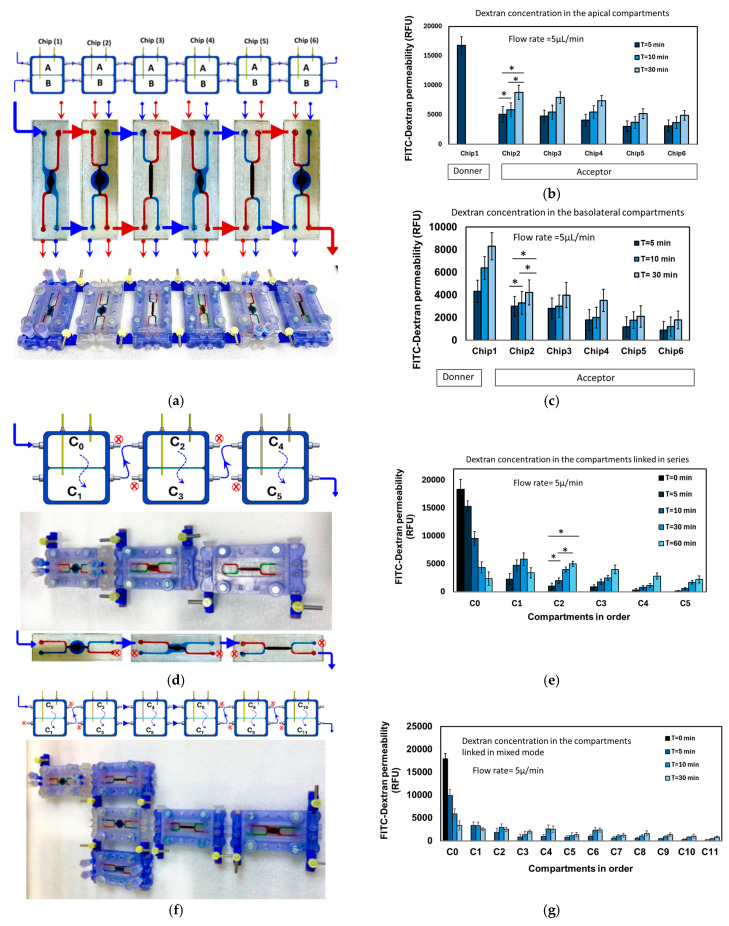
(**a**) Six chips connected in parallel where all the apical compartments are linked together by the two-way valves and, similarly, the basolateral compartments are linked together. Side view (top panel), top view (middle panel), and assembly image (bottom panel). The tracer transport profile within the downstream apical compartments (**b**) and basolateral compartments (**c**). The concentration profile exhibits a gradual decline in the downstream compartments. (**d**) Three chips are interconnected in series. In this arrangement, the basolateral compartment of the first chip is connected to the apical compartment of the next chip. (**e**) The concentration profile exhibits a gradual decline in the downstream compartments. (**f**) A complex assembly with six chips is interconnected in a mix mode, combining both series and parallel connections. (**g**) The corresponding tracer transport profile. * *p* < 0.1.

Each chip has four fluidic ports (two inlets and two outlets). Every fluidic port is accessed by two fluidic ports (channels) in the base unit (Figure 2e,f). The chip is inserted into the base unit where the four holes in the chip are aligned to four hollow protruding channels in the base unit. To ensure fully sealed connection, an elastic rubber gasket which was 3D printed was inserted beneath the chip. Then, the chip was tightly fixed using a cover with four screws (Figure 2e,f). The cover was secured onto the chip using four screws, ensuring a tight connection between the chip and the base unit. Chip-to-chip connecting can be established in three different configurations: (1) in parallel: in this configuration, the upper chambers are connected, and the lower chambers are also connected together (Figure 3a); (2) in series: the lower chamber of the first chip is connected to the upper chamber of the next chip (Figure 3d); (3) complex connection: a set of chips are connected in mix mode (i.e., both in parallel and in series) (Figure 3f).

The fluidic ports of the first unit were connected to the culture media reservoir through a set of PEEK tubing with an inner diameter of 0.5 mm and the ports of the last unit were connected to a set of syringes which were mounted on a programmable syringe pump (KDScientific, Holliston, MA, USA). The entire perfusion system, except the syringe pumps, was kept in the CO_2_ incubator and negative pressure was applied onto the syringes to provide continuous perfusion of cell culture media through the cell culture chambers at a flow rate of 5–10 nL/s. To characterize chip–chip communication, chips were connected in various chip–chip connections (Figure 2) and flow of colored liquid was monitored.

### 2.5. Characterization of the Chip-to-Chip Crosstalk

An arbitrary number of chips were interconnected with the selected connection scheme and the transport of colored deionized water (DI) was observed through the chips. The ability of the integrated valves to control/divert the fluid flow was examined for various chip assemblies. In addition, the transport of the tracer FITC-dextran (4K Da) through the porous membranes was measured. The tracer solution with a concentration of 10 µL/mL was injected in the upper chamber of the upstream chip, which was initially filled with phosphate-buffered saline (PBS) solution, and the fluorescence intensity due to the diffusion of the tracer was measured at different downstream check points using a fluorescent plate reader (Perkin Elmer, Richmond, CA, USA). The side ports were utilized for injection and sampling, as illustrated in Figure 2e. These ports remain closed under normal conditions and are opened only during injection or sample collection.

### 2.6. System Characterization and Cell Culture

Small intestine in vitro model: To construct a simplified in vitro model of the epithelium of the human small intestine, a confluent layer of Caco-2 cells (American Type Culture Collection (ATCC), Manassas, VA, USA) was grown on top of the porous membrane. Caco-2 cells were initially grown on 25 mL tissue culture flasks in DMEM/F-12 cell culture media with high glucose and with 10% (*v*/*v*) fetal bovine serum (FBS) and 1% penicillin/streptomycin antibiotic at 37 °C, 5% CO_2_, in 95% relative humidity till reaching 80% confluence. Culture medium was refreshed every 24 h hours and cells were harvested by passaging Trypsin/EDTA solution.

The microfluidic chips were sterilized with 70% ethanol and exposed to UV light for 1 h. Then, the chips were washed with deionized (DI) water and subsequently, a volume of 300 µL of a Poly-L-lysine solution (0.1 mg/mL, in H_2_O) was loaded into the chip and incubated overnight at 37 °C. Afterward, the chips were washed with sterilized DI water and PBS using a pipette. The apical and basolateral chambers of the chip were filled with fresh culture media and incubated for 24 h. Then, the medium in the apical chamber was replaced with Caco-2 cell suspension at a concentration of 2–3 × 10^5^ cells/mL. The medium in the basolateral chamber was also replaced with a fresh one. The chips were then placed inside the CO_2_ incubator with perfusion flow switched OFF for 24 h to allow cell attachment. Then, the perfusion was switched ON as described above at a flow rate of 10 nL/s.

***Cell viability***: To assess cell viability, a mixture of calcein-AM (2 M) and ethidium homodimer-1 (4 M) was prepared and diluted in Dulbecco’s Phosphate Buffered Saline (D-PBS). The mixture was then introduced into the cell compartment and allowed to incubate for a duration of 10 min. Then, the cell viability was examined using a fluorescent microscope. Cell viability was calculated as the percentage of calcein-AM-labeled cells. Images were captured from various locations within the cell compartment and the percentage values obtained from these images were then averaged. Data were represented as mean ± SD.

### 2.7. Immunofluorescence Staining of Intercellular Tight Junctions

Once a confluent monolayer of the Caco-2 cells was established, the formation of the intercellular TJs in the Caco-2 monolayer was detected by immunofluorescence staining. The membrane with the cells was detached from the chip and placed on a cover slip. The cells were washed with PBS and fixed with 4% paraformaldehyde (PFA) (Catalog #P6148, Sigma, Burlington, MA, USA) and incubated for 15 min. Then, the cells were permeabilized with 0.25% Triton X-100 (Catalog #X100, Sigma) in PBS and incubated for 10 min followed by washing with PBS. Non-specific binding sites were blocked with 5% bovine serum albumin (BSA) (Catalog #A2153, Sigma) in PBS for 30 min. The sample was then incubated with 2 µg/mL anti-mouse occludin antibody (Catalog# 331500, Life Technologies Carlsbad, CA, USA) overnight at 4 °C. The sample was then washed with PBS and treated with 3 µg/mL anti-mouse IgG conjugated to FITC (Catalog# 339111, Life Technologies) for 1 h in the dark at room temperature. The samples were then washed with PBS. The cells were inspected using a fluorescent microscope (CKX53, Olympus, Tokyo, Japan) equipped with a high-resolution charge-coupled device (CCD) camera (DP73, Olympus).

### 2.8. TEER Measurements

Prior to inserting into the chips (Figure 2e), the gold wires were sterilized with isopropanol (IPA), rinsed with PBS, dried with air jet, and irradiated with UV light. The other ends of the wires were then connected to a digital multimeter (BK Precision, Yorba Linda, CA, USA) to measure the TEER. One chip without cells was used as a control. TEER measurements were taken at regular intervals throughout the experiment. The data points were obtained by averaging three measurements. The contribution of the cells was estimated by subtracting the electrical resistance of the porous membranes without cells from the total measured values and normalized by multiplying the measured electrical resistance by the total surface area of the cell layer to calculate TEER in Ω/cm^2^.

### 2.9. Trans-Epithelial Permeability

The permeability of FITC-dextran (4K Da) was measured through the epithelial layers at various check points. Initially, chips which are placed in the base units were filled with cell culture medium and placed in the CO_2_ incubator for at least 1 h. An amount of 10 µL of the FITC-dextran solution with a concentration of 10 g/mL was injected into the donner compartment of the chip (e.g., the apical compartment of chip 1) and samples were serially collected from various downstream acceptor compartments with different chip assemblies at different time intervals. The concentration of the tracer molecule which is proportional to the trans-epithelial flux was quantified using a fluorescent plate reader (Perkin Elmer, Richmond, CA, USA). The associated TEER values with sampling were also measured.

### 2.10. Immune Cell Culture

The THP-1 cell line (TIB-202, ATCC; obtained from Sigma-Aldrich) was utilized as a model for human immune-responsive monocytes and macrophages. The cells were initially cultured in RPMI-1640 medium and subsequently adapted to DMEM medium supplemented with 10% fetal bovine serum, 1% penicillin, and 1% L-glutamine to enable co-culture with Caco-2 cells. The cell culture procedure for THP-1 cells was similar to that used for Caco-2 cells. THP-1 cells, at a concentration of 5 × 10^4^ cells/mL, were inoculated into the upper chamber using a 5 mL syringe through a dedicated fluidic port. Since the cells remain in suspension and do not adhere to surfaces, the inlet and outlet of the upper chamber were blocked after inoculation to prevent cell loss during the experiment. Cell culture medium was perfused from the lower chamber through the porous membrane. To induce differentiation of monocytic cells into adherent macrophages, THP-1 cells were treated with 200 nM of phorbol 12-myristate 13-acetate (PMA) and incubated in a CO_2_ incubator for 24 h. Non-adherent cells were aspirated, and the adherent cells were washed with PBS. Differentiation was confirmed by microscopic inspection, with differentiated cells (macrophages) adhering to the porous membrane following PMA treatment.

## 3. Results and Discussions

### 3.1. Characterization of the Fluidic Routing Through the Chip Assemblies

The primary objective of this study is to establish a modular microfluidic network that serves as a versatile platform for cell co-culture, accommodating various cell-based biological models. This is achieved by constructing a complex fluidic network through the connection of two or more 3D microfluidic chips using different connecting schemes. The base unit, which is 3D printed, serves as the host for a double-layered chip and allows for the receipt and transfer of fluids in different directions through a set of valves. Multiple base units can be interconnected seamlessly and securely using 3D printed fitting connectors, as illustrated in Figure 2g. To characterize the movement of fluids through the compartments and permeable membranes in this complex system, several microchips were linked together utilizing three distinct connecting configurations (Figure 3a,d,f). The transport and diffusion of fluorescently labeled dextran molecules (FITC-dextran) with a molecular weight of 4K Da was then monitored and quantified along specific paths. This was conducted without incorporating any living cells into the system. Figure 3a shows six chips connected in parallel where all the apical compartments are linked together by the two-way valves and, similarly, the basolateral compartments are linked together. The tracer solution was injected into the apical compartment of D1 (donner compartment) and the subsequent fluorescence intensity (FI) in the downstream compartments (acceptor compartments), which correlates with the concentration of the tracer, was measured (Figure 3b,c). The concentrations of the tracer within the various compartments offer a quantitative measure of the transport and distribution of the tracer solution in the interconnected microfluidic system. Figure 3b shows the profile of the tracer concentration in the apical compartments. The initial concentration in the donner compartment is high, and the subsequent concentration decreases gradually in the downstream compartments. The concentration increases with time during the measurement within 30 min. Similarly, the concentration profile within the basolateral compartments also exhibits a gradual decline in the downstream compartments (Figure 3c).

Figure 3d shows the configuration where three chips are interconnected in series. In this arrangement, the basolateral compartment of the first chip is connected to the apical compartment of the next chip, and this alternating pattern continues. The tracer solution diffuses through the membrane from the apical compartment to the basolateral compartment in each chip. Following the same pattern as above, the concentration profile of the tracer increases with time and gradually decreases along the downstream compartments. Figure 3f presents a complex assembly where six chips are interconnected in a mix mode, combining both series and parallel connections. This configuration, better illustrated in the top panel, demonstrates the flexibility of connecting the apical compartment of a specific chip to either the apical or basolateral compartments of the subsequent chip. Figure 3g displays the corresponding tracer transport profile, which follows a similar pattern as described in the previously mentioned assemblies. These observations serve as evidence for the effective interconnectivity of fluid communication between a multitude of fluidic compartments that are arranged in a three-dimensional architecture. It is important to note that the connection or disconnection of any chip within the complex assembly can be accomplished by manipulating the valve set without causing any interruption to the flow in the other chips. This procedure entails deactivating the corresponding valve, inserting or detaching the desired chip, and subsequently reactivating the valves to ensure the resumption of fluid flow. Moreover, it is important to highlight that any selected chip within the assembly can be disconnected from the fluidic system and subsequently reconnected using the valves, while still remaining part of the overall assembly. For instance, if three chips are connected in series, the first chip can be fluidically disconnected by switching the valve between chip 1 and chip 2. In this configuration, chips 2 and 3 remain connected, while chip 1 operates independently. Similarly, the chip in the middle (chip 2) can be disconnected, leaving chips 1 and 3 linked together by bridging the two chips with dedicated tubing. In future studies, a more advanced valving system will be implemented to simplify multi-mode connectivity. In summary, the platform facilitates a seamless and flexible chip assembly process, allowing for fluid switching and redirection across different spatial and temporal dimensions.

### 3.2. Multi-Intestinal Epithelium System

The intestinal epithelium and absorption are modeled in vitro using a monolayer intestinal cell line such as Caco-2 and HT-29 [27,28]. Specifically, the Caco-2 cell culture has been widely utilized to predict apparent permeability coefficients of drugs based on intestinal barrier properties [29,30,31]. This colon cancer cell line undergoes differentiation to acquire a morphology resembling that of the human small intestine, characterized by brush border microvilli that project vertically above the monolayer’s surface [32]. The monolayer develops asymmetrically and harbors enzymes linked to the brush border and TJs [33]. Cells grown on microporous membranes exhibit an apical surface orientated towards the upper compartment and a basolateral surface facing the lower compartment [32].

**Figure 4:**
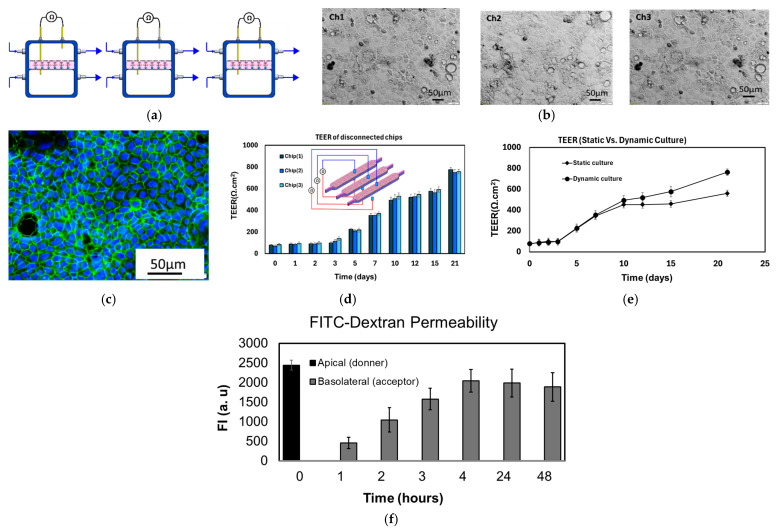
(**a**) Caco-2 cells were seeded in three identical chips with no interconnection. (**b**) Optical image of the confluent monolayers within the three chips. (**c**) TJs formed by adjacent Caco-2 cells. The TJs were stained by FITC-labeled anti-mouse occluding. Images of confluent monolayer formed by Caco-2 cells taken on day 21 under 10× objective lens. (**d**) TEER values increased progressively over time with a substantial increase observed after 5 days, indicating the presence of the tight junction proteins. (**e**) Perfusion accelerates the TJ formation and maintains higher TEER value after the formation of the confluent layer. (**f**) The fluorescence intensity, which corresponds to the concentration of the tracer, exhibited a gradual increase over the 48 h duration.

Three disconnected systems: Caco-2 cells were cultured in three separate chips, each functioning independently and not connected to each other, as shown in Figure 4a. The cells were seeded on top of porous membranes and maintained in a CO_2_ incubator under dynamic culture conditions, with a flow rate of 10 nL/s. The proliferation and differentiation of the cells were closely monitored until fully differentiated enterocyte monolayers were formed. In this particular configuration, three epithelial layers, referred to as EP1, EP2, and EP3, were created. For this experiment, D3 was utilized, as illustrated in Figure 1c. Fully confluent monolayers of Caco-2 cells were observed after 7–10 days of culture in the three chips (Figure 4b). The formation of a fully confluent monolayer that covers the entire membrane is crucial for evaluating the Caco-2 layer as a robust model of the intestinal barrier, particularly for conducting permeability assays. The presence of TJs, specifically occludin, was observed after a 21-day culture period (Figure 4c). The trans-epithelial electrical resistances (TEERs) were independently measured in the three chips over a span of three weeks (Figure 4d). It was observed that the TEER values increased progressively over time with a substantial increase observed after 5 days, indicating the presence of the TJ proteins.

To investigate the impact of perfusion flow on the integrity of the Caco-2 monolayer and the formation of TJs, a flow was introduced to two chips (through both the apical and basolateral compartments). Meanwhile, the third chip was maintained under static conditions, with regular manual media changes every 24 h. The TEER was measured concurrently in all three chips. As illustrated in Figure 4e, the TEER values obtained from the perfusion-based culture demonstrated higher values compared to those observed in the static system, particularly after the establishment of a fully confluent layer (i.e., 1 week after cell seeding). The permeability of FITC-dextran (4K Da) from the apical to basolateral compartment was investigated over a two-day period under dynamic flow conditions. The tracer was introduced into the apical compartment, and samples were collected from the basolateral compartment at various time intervals. The fluorescence intensity (FI), which corresponds to the concentration of the tracer, exhibited a gradual increase over the 48 h duration (Figure 4f).

**Figure 5:**
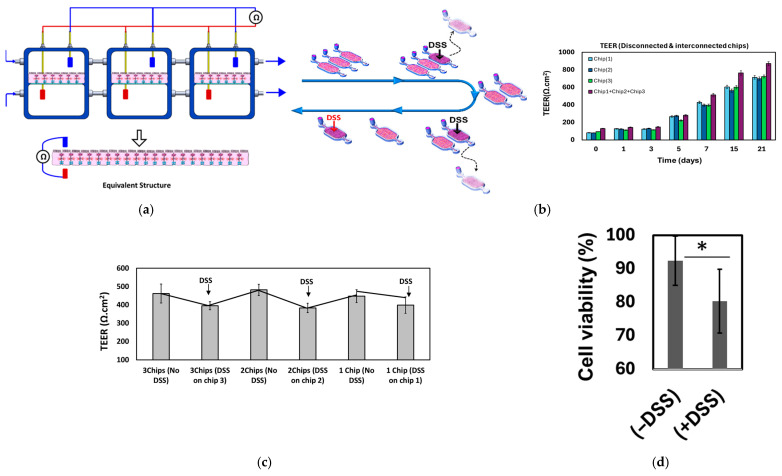
(**a**) The experimental design involved a multi-epithelium structure consisting of three epithelial model chips that were fluidically connected in parallel. The apical compartments of the chips were connected, as were the basolateral compartments. Chip 3 was treated with DSS (5k Da (2%)), and its TEER was examined. Subsequently, chip 3 was detached, and the TEER was assessed again. Following this, chip 2 was subjected to DSS treatment, and its TEER was inspected. Finally, chip 2 was detached, and the TEER for it and for chip 1 were measured. (**b**) The TEER values for every individual monolayer (EC1, EC2, EC3) and when they are connected (EC1+EC2+EC3) over a 15-day culture period. Both individual layers and connected layers exhibit an increase in TEER over time. While the TEER values for the individual layers are comparable, the TEER of the connected layers slightly surpasses that of the individual layers, notably after one week of culture. (**c**) When one layer was treated with DSS, the total TEER dropped to approximately 80% of its original value compared to the untreated layer. Upon disconnection/removal of the treated monolayer, the TEER returned to its initial level. Consequently, the TEER values of one, two, or three healthy monolayers were found to be comparable. (**d**) Cell viability drops upon treating the cells with DSS. * *p* < 0.05.

Three systems connected in parallel: Three cell monolayers were grown in three identical chips that were connected in parallel, as illustrated in Figure 5a. Specifically, the three apical compartments were connected, and likewise, the basolateral compartments were connected. In addition, the three electrodes on the apical sides were linked, as were the three electrodes on the basolateral sides. The cells were monitored to ensure the establishment of fully confluent monolayers in all three chips. To ensure the stability of the epithelial monolayer, the TEER was continuously monitored for a duration of 15 days. The TEER values exhibited stability and demonstrated close readings across the three individual chips, with a noticeable increase over time. The overall total TEER also followed a similar trend. Figure 5b shows the TEER values for every individual monolayer (EC1, EC2, EC3) and when they are connected (EC1 + EC2 + EC3) over a 21-day culture period, which have been normalized to the surface area of the epithelial layer in Ω.cm^2^. Individual monolayers and connected monolayers exhibit an increase in TEER over time. While the TEER values for the individual monolayers are comparable, the TEER of the connected monolayers slightly surpasses that of the individual ones, notably after one week of culture. This may be attributed to the larger volume of cell culture media present when the three chips are connected and the variations in ion transport compared to the individual monolayer setup.

Dextran sulfate sodium (DSS) is widely used in inducing colitis in animal models, which is associated with decreased intestinal barrier function and rapid damage to the intestinal epithelium [34,35]. After 20 days of cell seeding, the cell monolayers were treated with 100 µL of DSS according to the following sequence, as illustrated in Figure 5a: (i) initially, the three healthy, fully confluent monolayers of epithelial cells (EC1, EC2, EC3) within three distinct chips were connected in parallel, and the total TEER value was measured. (ii) EC3 was treated with DSS (2%), and the total TEER was measured. (iii) EC3 was disconnected (removed), and the total TEER was measured for EC1+EC2. (iv) EC2 was treated with DSS (2%), and the total TEER was measured. (v) EC2 was disconnected, and the TEER was measured for EC1. (vi) EC1 was treated with DSS (2%), and the TEER was measured. TEER measurements were conducted 24 h after DSS treatment and normalized to the epithelial layer surface area to provide the resistance per unit area, as shown in Figure 5b. Figure 5c shows the measured TEER following the aforementioned protocol. When one layer was treated with DSS, the total TEER dropped to approximately 80% of its original value compared to the untreated layer. Upon disconnection/removal of the treated monolayer, the TEER returned to its initial level. Consequently, the TEER values of one, two, or three healthy monolayers were found to be comparable. This experimental configuration, utilizing multiple identical monolayers, has the potential to facilitate prolonged and multi-parameter studies within the same cell culture. Treating the cells with DSS also affected cell viability, leading to a decrease of approximately 20% after DSS treatment (Figure 5d).

**Figure 6:**
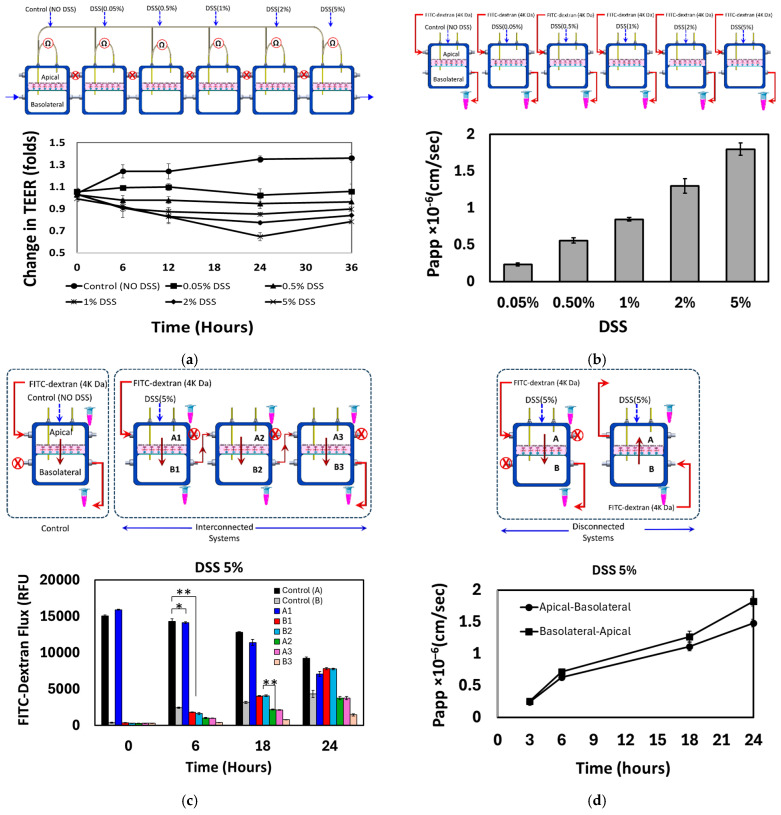
The effect of DSS at various concentrations (0.05–5%) on the integrity of the Caco-2 monolayer (TEER). (**a**) Six Caco-2 monolayers were cultivated on separate chips. Upon reaching confluency, the basolateral compartments of these six chips were interconnected, creating a single large compartment that linked the six apical compartments through the membrane. TEER values exhibited a significant decrease after 24 h of DSS treatment, particularly at higher concentrations of DSS (i.e., 2% and 5%). (**b**) The EC monolayers were disconnected and the apical-to-basolateral transport (permeability) of FITC-dextran (4 kDa) was measured. The permeability of the EC increases with increasing DSS concentrations. (**c**) Three chips with fully confluent monolayers were connected in series. The concentration of FITC-dextran decreases in the donor compartment and gradually increases in the downstream compartment. However, the concentration is observed to be maintained at a high value in the chambers along the transportation path during the experiment. (**d**) The basolateral–apical transport of FITC-dextran was greater than that of apical–basolateral when examined using two individual disconnected chips. * *p* < 0.05, ** *p* < 0.01.

***Six basolateral connected systems***: The impact of DSS treatment at various concentrations on the EC layers’ integrity and permeability was evaluated. Six chips containing identical Caco-2 cell monolayers were interconnected through basolateral compartments following the establishment of fully confluent and differentiated monolayers (Figure 6a (top panel)). The electrodes accessing the basolateral compartments were also interconnected. The monolayers (EC1, EC2, EC3, EC4, EC5, EC6) in these chips were exposed to DSS at concentrations of 0%, 0.5%, 1%, 2%, and 5%, respectively. The TEER of each monolayer was assessed individually by linking the apical electrode of each chip with the common basolateral electrode. Figure 6a shows that DSS treatment leads to a reduction of the measured TEER for each individual EC monolayer. Next, the six chips were disassembled, and the apical-to-basolateral transport (permeability) of FITC-dextran (4 kDa) through each monolayer was measured, as illustrated in Figure 6b (top panel). The permeability of the EC monolayers was observed to increase with increasing DSS concentrations (Figure 6b). These results provide evidence that exposure of the EC monolayers to DSS causes TJ damage, epithelial barrier dysfunction, and increased intestinal permeability. Treatment with DSS was shown to induce the expression of phospholipase D2 (PLD2) and downregulate the TJ occludin in colon epithelial cells. PLD2 was found to mediate the phosphorylation and proteasomal degradation of occludin, leading to disruption of the TJs [36,37]. The loss of the TJ protein ZO-1 has been also observed in a DSS-induced colitis mouse model [38]. Altered or compromised expression of epithelial TJ proteins is considered a key factor in the pathogenesis of inflammatory bowel diseases like ulcerative colitis, which can be induced by DSS in animal models.

Three systems connected in series: To examine the transport of dextran molecules through multi-EC monolayers, three chips with fully confluent monolayers were connected in series such that the basolateral compartment of the first chip is connected to the apical compartment of the second chip and the basolateral compartment of the second chip is connected to the apical compartment of the third chip. The transport pathway involved the following sequence: apical (1)→basolateral (1)→apical (2)→basolateral (2)→apical (3)→basolateral (3). Within this arrangement, the tracer molecules will be transported across three EC monolayers in the apical-to-basolateral direction. During the 24 h period, the concentration of FITC-dextran decreases in the donor compartment and gradually increases in the downstream compartments. However, the concentration is observed to be maintained at a high value in the chambers along the transportation path during the experiment. The basolateral–apical transport of FITC-dextran was greater than that of apical–basolateral when examined using two individual disconnected chips (Figure 6d). Epithelial cells have a polarized organization, with different apical and basolateral domains separated by TJs. This organization created a barrier that restricts the movement of molecules between the apical and basolateral compartments [39]. Moreover, epithelial cells express different transporters and channels on their apical and basolateral surfaces. This asymmetric distribution of transporters facilitates the preferential movement of molecules in the basolateral-to-apical direction [40].

### 3.3. EC–Immune Cell Co-Culture and Cytokine Expression Measurements

**Figure 7:**
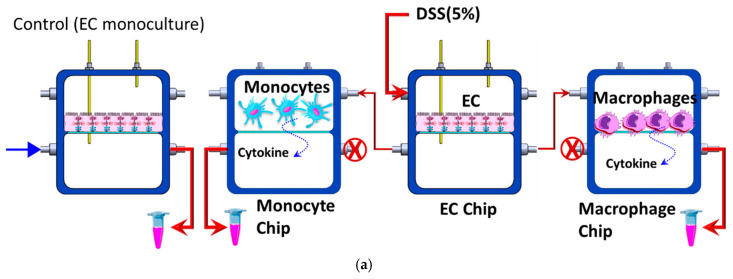

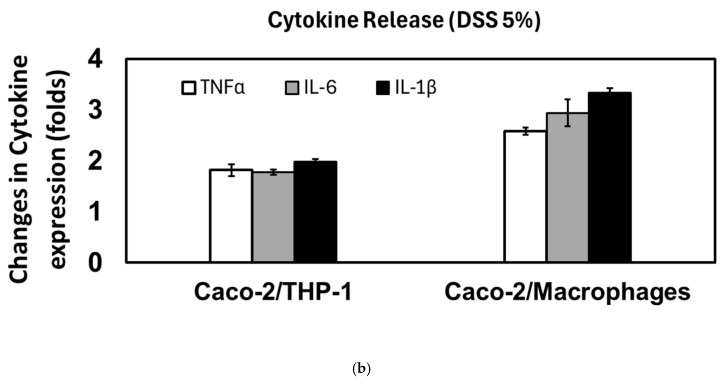
(**a**) The experimental design of EC–immune cell co-culture and stimulation. THP-1 monocytic cells were seeded within two separate chips, as illustrated in Figure 7a. The THP-1 cells within one chip were treated with PMA to induce the differentiation of the cell to adherent macrophages. The THP-1 cells in the other chip (monocyte chip) were kept untreated. An EC monoculture was used as a control setup. (**b**) The expression of cytokines in the Caco-2/THP-1 and Caco-2/macrophage co-cultures was evaluated relative to levels seen in control Caco-2 epithelial cell monocultures. Macrophages secreted elevated amounts of cytokines when exposed to DSS compared to the THP-1 monocytes. The numbers of THP-1 and macrophage immune cells incorporated into the co-cultures were maintained at comparable levels.

Caco-2 cells were cultured on a porous membrane on one chip (EC chip) and maintained until reaching confluency and maturation. Equal numbers of THP-1 monocytic cells were seeded within two separate chips, as illustrated in Figure 7a. The THP-1 cells within one chip were treated with 200 nM of phorbol 12-myristate 13-acetate (PMA) and incubated in the CO_2_ incubator for 24 h to induce the differentiation of the monocytic cells to adherent macrophages. The undifferentiated monocytes appeared rounded in shape and remained suspended while the differentiated macrophages appear to adhere to the porous membrane after the treatment with PMA. Subsequently, the non-adherent cells were aspirated. The THP-1 cells in the other chip (monocyte chip) were kept untreated (Figure 7a). An EC monoculture was used as a control setup. The three chips were interconnected so that the basolateral of the EC chip was connected to the apical chambers of the monocyte chip and macrophage chip where the cells were present. The basolateral chambers of the immune cell chips were used for sampling to prevent cell disturbance.

The supernatants were collected from the lower/basolateral compartments and the expression of three pro-inflammatory cytokines (TNFα, IL-6, and IL-1β) was measured using ELISA, as explained elsewhere [41]. Figure 7b shows the levels of expression of these cytokines compared to those obtained from the EC monoculture. Macrophages released a higher level of cytokines upon DSS treatment as compared to monocytes which suggests that macrophages are more sensitive to DSS. Macrophages can polarize into pro-inflammatory M1 or anti-inflammatory M2 phenotypes in response to stimulation. DSS exposure likely drives macrophages towards a more inflammatory M1 phenotype, leading to heightened sensitivity and production of inflammatory cytokines like IL-1β and TNF-α. In contrast, monocytes may not undergo the same degree of polarization or inflammatory activation in response to DSS [42,43].

## 4. Conclusions

The integrated system with multiple chips’ interconnection demonstrates significant potential in advancing organ-on-a-chip technology. The establishment of a versatile platform for cell co-culture, fluid transport assessment, and complex chip assembly showcases the adaptability and effectiveness of the system. The ability to manipulate fluid flow seamlessly and flexibly across various spatial and temporal dimensions highlights the utility and promise of this integrated approach. The system was fabricated using low-cost materials and affordable tools (i.e., laser cutting and 3D printing), which enables high throughput disposable chip fabrication and a reliable system. The fluid flow and mass transport were investigated by monitoring the tracer FITC-dextran through different assemblies. To demonstrate the utility of the system for studying the cell–cell or tissue–tissue interaction, multi-monolayers of intestinal epithelial cells were used as the model. EC monolayers were grown in multiple chips and interconnected in various configurations. TEER and permeability profiles were investigated in detail for these configurations upon treatment of the cells with DSS. Also, the immune cells co-culture was stimulated through the ECs and the expression of inflammatory cytokines was detected. This miniaturized platform offers controlled conditions for co-culturing key cellular components and assessing potential therapeutic agents in a physiologically relevant setting. Future research building upon these findings could further exploit the system to establish multi-organs-based in vitro models, enhancing the capabilities and applications of organ-on-a-chip technology in drug discovery and development.

## Data Availability

The original contributions presented in this study are included in the article. Further inquiries can be directed to the corresponding authors.
